# SIRT3 activation by oroxylin A phosphate diethyl ester triggers mitochondrial dysfunction and apoptosis in non-small cell lung cancer

**DOI:** 10.7150/ijbs.133993

**Published:** 2026-05-11

**Authors:** Chiang-Wen Lee, Yu-Feng Lin, Kuan-Han Lee, Ying-Sui Sun, Tsung-Ming Chang, Miao-Ching Chi, Zih-Chan Lin, Ayaho Yamamoto, Kuo-Ti Peng, Ju-Fang Liu

**Affiliations:** 1 Department of Respiratory Care, Chang Gung University of Science and Technology, Chiayi County 61363, Taiwan.; 2 Chronic Diseases and Health Promotion Research Center and Center for smart healthcare and Drug Research and Development, Chang Gung University of Science and Technology, Chiayi County 61363, Taiwan.; 3 Department of Orthopaedic Surgery, Chang Gung Memorial Hospital, Chiayi County 61363, Taiwan.; 4 Department of Safety Health and Environmental Engineering, Ming Chi University of Technology, New Taipei City 243303, Taiwan.; 5 Department of Medical Laboratory Science and Biotechnology, Asia University, Taichung 41354, Taiwan.; 6 School of Pharmacy, Tzu Chi University, Hualien 970374, Taiwan.; 7 School of Dental Technology, College of Oral Medicine, Taipei Medical University, Taipei 11031, Taiwan.; 8 Division of Pulmonary and Critical Care Medicine, Chang Gung Memorial Hospital, Chiayi 61363, Taiwan.; 9 Chronic Diseases and Health Promotion Research Center, Chang Gung University of Science and Technology, Chiayi 61363, Taiwan.; 10 Child Health Research Centre, The University of Queensland, South Brisbane, Queensland 4101, Australia.; 11 Department of Orthopedic Surgery, Chang Gung Memorial Hospital, Puzi City, Chiayi County 61363, Taiwan.; 12 Graduate Institute of Clinical Medical Sciences, College of Medicine, Chang Gung University, Taoyuan 333323, Taiwan.; 13 School of Oral Hygiene, College of Oral Medicine, Taipei Medical University, Taipei 11031, Taiwan.; 14 Department of Medical Research, China Medical University Hospital, China Medical University, Taichung 40447, Taiwan.; 15 Translational Medicine Center, Shin Kong Wu Ho-Su Memorial Hospital, Taipei 111045, Taiwan.

**Keywords:** lung cancer, SIRT3, mitochondrial dysfunction, OA-OEt, mitochondrial fission

## Abstract

Lung cancer remains the leading cause of cancer mortality worldwide, and outcomes for advanced non-small cell lung cancer (NSCLC) are limited by resistance and toxicity. Sirtuin 3 (SIRT3), a mitochondrial NAD^+^-dependent deacetylase, regulates mitochondrial homeostasis and redox balance, but its therapeutic potential in NSCLC is incompletely defined. Here, bioinformatic analyses, structure-based virtual screening, and *in vitro* and *in vivo* validation were used to evaluate SIRT3 modulation in NSCLC. Although SIRT3 mRNA expression varied across lung cancer cohorts, higher SIRT3 expression was associated with better overall survival in lung adenocarcinoma (LUAD), and protein-level analyses demonstrated reduced SIRT3 expression in lung cancer specimens. Immunohistochemical analysis of clinical specimens and western blotting of NSCLC cell lines further confirmed reduced SIRT3 expression in lung cancer. To identify a SIRT3-targeting compound, we performed structure-based virtual screening using the SIRT3 crystal structure and prioritized oroxylin A phosphate diethyl ester (OA-OEt), a derivative of oroxylin A. OA-OEt markedly enhanced SIRT3 activity and exhibited stronger antiproliferative effects than oroxylin A in H1299 and A549 cells. Mechanistically, OA-OEt suppressed cell growth, induced G2/M arrest, and increased apoptosis. OA-OEt disrupted mitochondrial homeostasis, including elevated mitochondrial superoxide, reduced SOD2, altered mitochondrial dynamics markers, and impaired mitochondrial respiration; mitochondrial ROS scavenging partially rescued apoptosis. In an H1299 xenograft model, OA-OEt reduced tumor growth without overt body-weight loss and increased tumor SIRT3 and cleaved caspase-3 with consistent mitochondrial marker changes. Collectively, these findings support SIRT3 as a functionally tumor-suppressive mitochondrial regulator in NSCLC and suggest that OA-OEt may represent a promising SIRT3-activating lead compound.

## Introduction

Lung cancer accounts for approximately 1.8 million deaths each year, with non-small cell lung cancer (NSCLC) comprising about 85% of all cases. Despite considerable advances in surgery, chemotherapy, radiotherapy, targeted therapy, and immunotherapy, the prognosis of advanced NSCLC remains dismal, with a 5-year survival rate of only 17.4% 1. Although targeted agents, including tyrosine kinase inhibitors (TKIs) and immune checkpoint inhibitors, have markedly improved clinical outcomes for subsets of patients harboring actionable mutations, their efficacy is often limited by acquired resistance, insufficient durability of response, adverse effects, and substantial costs 2, 3. These limitations underscore the urgent need to identify novel therapeutic targets and more effective treatment strategies.

Sirtuin 3 (SIRT3) is a mitochondrial NAD⁺-dependent deacetylase that regulates cellular metabolism, oxidative stress responses, and cell survival. It modulates critical processes such as the tricarboxylic acid cycle, fatty acid oxidation, and oxidative phosphorylation, thereby maintaining mitochondrial homeostasis and redox balance 4. Notably, SIRT3 exhibits context-dependent roles in cancer biology, acting as a tumor suppressor in some malignancies yet supporting tumor survival in others by sustaining mitochondrial function and metabolic adaptability 5, 6. However, its precise role and therapeutic potential in lung cancer remain poorly defined.

Mitochondrial dynamics, maintained by a balance between fission and fusion, are crucial for cellular adaptation to metabolic stress and mitochondrial quality control. Dysregulation of these processes contributes to tumorigenesis, metastasis, and resistance to cell death 7-10. Mitochondrial fission is regulated by proteins such as dynamin-related protein 1 (DRP1) and fission 1 (FIS1), whereas fusion is mediated by mitofusin 1/2 (MFN1/2) and optic atrophy 1 (OPA1). Targeting mechanisms that govern mitochondrial dynamics and associated redox vulnerabilities have emerged as a promising anticancer approach.

Oroxylin A (OA), a naturally occurring flavonoid derived from *Scutellaria* species, exhibits anti-inflammatory and anticancer activities in several cancer models 11, 12. Previous studies have shown that OA can enhance SIRT3 activity and alter mitochondrial metabolic programs in cancer cells 13, 14. However, its clinical translation is hampered by poor bioavailability and chemical instability 15. To address these limitations, we identified a modified derivative, oroxylin A phosphate diethyl ester (OA-OEt), designed to improve physicochemical stability and enhance SIRT3-activating potential.

In this study, our findings support SIRT3 as a functionally tumor-suppressive mitochondrial regulator with a favorable prognostic association in LUAD. Using a structure-based discovery approach, we analyzed OA-OEt, a SIRT3-activating compound, and examined its anticancer activity and mitochondrial mechanisms in NSCLC models *in vitro* and *in vivo*.

## Material and Methods

### Chemicals

Primary antibodies against Caspase-3, Caspase-9, PARP, SIRT3, Beclin-1, p62, LC3B, SOD2, DRP1, FIS1, OPA1, MFN2, and β-actin were purchased from GeneTex (Irvine, CA, USA). Antibodies for CDK1 and cyclin B1 were obtained from Merck Millipore (Burlington, MA, USA). Secondary anti-rabbit and anti-mouse antibodies were sourced from Santa Cruz Biotechnology (Dallas, TX, USA). The SIRT3 Activity Assay Kit was purchased from Abcam (ab156067, Cambridge, MA, USA). All other chemicals were obtained from Sigma-Aldrich (St. Louis, MO, USA).

### Cell culture

Human NSCLC cell lines H1299 and A549 were obtained from ATCC (Manassas, VA, USA) and the Bioresource Collection and Research Center (Hsinchu, Taiwan), respectively. H1299 cells were cultured in RPMI-1640 medium, and A549 cells in Ham's F-12 medium, both supplemented with 10% fetal bovine serum, 100 U/mL penicillin, and 100 μg/mL streptomycin. Cells were incubated at 37°C in a humidified atmosphere with 5% CO₂.

### Immunoblotting analysis

Cells were lysed in RIPA buffer, and equal amounts of protein were separated by SDS-PAGE and transferred to PVDF membranes (Immobilon, Merck Millipore). Membranes were blocked in 5% non-fat milk in TBST and incubated overnight at 4°C with primary antibodies (1:1000 dilution), followed by HRP-conjugated secondary antibodies (1:10,000 dilution) for 1 h at room temperature. Detection was performed using enhanced chemiluminescence and visualized with a UVP chemiluminescence detection system (Analytik Jena US, CA, USA).

### Synthesis and preparation of OA, OA-OEt, and OA-OH

**Scheme 1:**
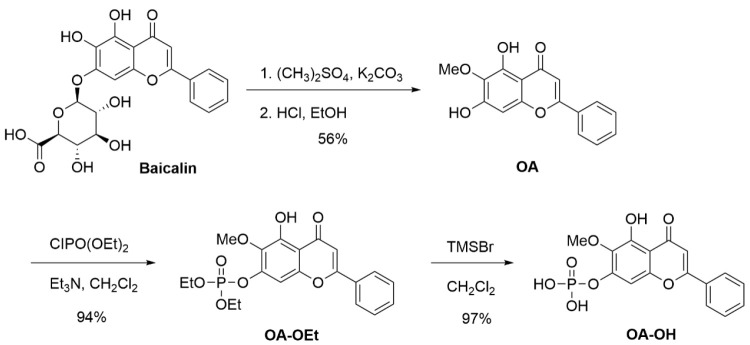
Synthetic procedure for OA, OA-OEt, and OA-OH. OA was synthesized from baicalin through methylation and deglucuronidation in a one-pot reaction, followed by derivatization to generate OA-OEt and OA-OH. All synthetic steps and methods have been patented (Process for preparing oroxylin A; US Patent 2019, US10239855B2).

OA was synthesized in 56% yield by methylating baicalin with dimethyl sulfate, followed by a deglucuronidation reaction with HCl-ethanol solution in a one-pot reaction. The brief synthetic pathway of OA-OEt and OA-OH is shown in Scheme 1. The successful synthesis of OA and its derivatives, OA-OEt and OA-OH, was confirmed using HPLC and NMR. All synthesis steps and methods have been patented (process for preparing oroxylin-A; US patent 2019, US10239855B2).

Synthesis of OA: Baicalin (223 mg, 0.50 mmol) was mixed with dimethyl sulfate (1.26 g, 10.0 mmol) and potassium carbonate (345 mg, 2.5 mmol). The mixture was stirred at room temperature for 24 h. A solution of HCl/ethanol (10 mL, HCl/ethanol = 1:9) was added, and the mixture was heated to 80 ℃ and stirred for 12 h. After cooling, the solution was concentrated, diluted in ethyl acetate, and washed with water. Concentrated and silica gel column chromatography yielded OA (80 mg, 56%).

Synthesis of OA-OEt: A solution of OA (142 mg, 0.50 mmol) in dichloromethane (5 mL) was treated with triethylamine (253 mg, 2.5 mmol) and diethyl chlorophosphite (129 mg, 0.75 mmol) at room temperature. The reaction mixture was stirred for 2 h and then diluted with ethyl acetate. The organic layer was washed with water (2 × 20 mL), dried over anhydrous Na₂SO₄, filtered, and concentrated under reduced pressure. The crude product was purified by column chromatography to afford OA-OEt (198 mg, 94% yield).

Synthesis of OA-OH: A solution of OA-OEt (0.1260 g, 0.30 mmol) in anhydrous dichloromethane (2 mL) was prepared. To this solution, trimethylsilyl bromide (0.3215 g, 7.0 mmol) was added at room temperature. The reaction mixture was stirred at room temperature for 8 hours. n-Hexane (5 mL) was added, leading to a precipitate. The precipitated solid was collected and dried to afford OA-OH as a yellow solid (0.1064 g, 97% yield).

### Molecular docking and binding pocket analysis

The crystal structure of human SIRT3 (PDB ID: 4JSR) served as the template for *in silico* docking studies 16. The binding pocket was defined by all residues localized within an 8 Å radius of the co-crystallized ligand (1NQ) 17. A library of compounds from the NCI database (n = 279,156) was initially screened based on Lipinski's rule of five; subsequently, the filtered molecules were docked using iGEMDOCK, where binding affinities were evaluated via van der Waals, electrostatic, and hydrogen-bonding potentials 18, 19. The top 1,000 ranked compounds were further subjected to the SiMMap server to elucidate critical binding anchors and pharmacophoric moieties 20, 21. To validate their respective binding poses, OA (PubChem CID: 5320315) and its derivative, OA-OEt, were explicitly docked into the SIRT3 binding site. Finally, the intermolecular interactions between SIRT3 and OA/OA-OEt were characterized using the Protein-Ligand Interaction Profiler (PLIP) 22.

### SIRT3 activity assay

Lung cancer cells were treated with OA, OA-OEt, or OA-OH for 24 h and lysed to prepare enzyme samples. SIRT3 activity in cell lysates was measured using the SIRT3 Activity Assay Kit (Abcam, ab156067, Cambridge, UK) according to the manufacturer's instructions for quantification of SIRT3 activity in lysates. Briefly, assay buffer, fluorogenic substrate peptide, NAD⁺, and developer were added to black 96-well plates, and reactions were initiated by adding cell lysate (enzyme sample). Fluorescence was recorded every 1-2 min for 30-60 min (Ex 340-360 nm; Em 440-460 nm), and activity was calculated from the initial linear reaction velocity after subtraction of the no-enzyme control.

### Cell viability assay

Cells were seeded in 48-well plates at 1 × 10⁴ cells per well and allowed to attach overnight. Cells were then treated with the indicated concentrations of OA, OA-OEt, or OA-OH for 24 h. Cell viability was assessed using the CCK-8 assay (Sigma-Aldrich), and absorbance at 450 nm was measured with a spectrophotometer (BioTek, Winooski, VT, USA).

### Chromatin condensation analysis

Cells were treated with OA-OEt (3-30 µM) for 24 h, fixed with paraformaldehyde, and stained with 1 µg/mL DAPI (Merck Millipore) for 5 minutes. Nuclear morphology was observed using a fluorescence microscope (Nikon Eclipse Ti, Tokyo, Japan).

### Colony formation assay

Cells (1.5 × 10⁵) were seeded into 6-well plates and treated with OA-OEt (3-30 µM) for 6 h. After drug removal, 1 × 10³ cells were reseeded in OA-OEt-free medium and cultured for 14 days with medium changes every 3 days. Colonies were fixed, stained with 0.05% crystal violet, and solubilized with 33% acetic acid for absorbance measurement at 550 nm.

### Cell cycle analysis

Cells (5 × 10⁵) were seeded in 6-well plates and treated with various concentrations of OA-OEt for 24 h. Cells were harvested, stained with a PI solution (0.1% Triton X-100, RNase A 0.2 mg/mL, PI 10 μg/mL; Sigma-Aldrich, St. Louis, MO, USA), and analyzed using a flow cytometer (Accuri C5). Cell cycle distribution was determined using appropriate analysis software.

### Analysis of apoptotic and necrotic cells

Following 24 h treatment with OA-OEt, apoptotic and necrotic cells were identified using annexin V/propidium iodide (PI) assays (Sigma-Aldrich, St. Louis, MO, USA). Live cells were harvested and stained according to the manufacturer's instructions with 1 μg/mL PI and 0.5 μg/mL FITC-conjugated annexin V. Staining was performed in the dark at room temperature for 15 minutes, followed by analysis using a flow cytometer (Accuri C5).

### Transmission electron microscopy analysis

H1299 cells were treated with OA-OEt (30 μM) for 8 h and fixed in Karnovsky's fixative at 4°C, followed by standard processing for transmission electron microscopy. Images were acquired using a JEOL JEM-1400 microscope (Tokyo, Japan) to evaluate mitochondrial ultrastructural changes.

### Immunofluorescence analysis

Cells were fixed, permeabilized, and incubated with anti-p62 primary antibody (1:200) overnight at 4°C. After washing, cells were incubated with DyLight488-conjugated goat anti-rabbit IgG secondary antibody for 1 h. Nuclei were counterstained with DAPI, and images were captured using a fluorescence microscope.

### Mitochondrial superoxide and morphology analysis

Cells were treated with OA-OEt (3-30 μM) and stained with MitoSOX™ Red (Thermo Fisher Scientific) (5 μM) for 10 min to assess mitochondrial superoxide by flow cytometry. For mitochondrial morphology, live cells were stained with MitoTracker™ Green FM (M7514, Thermo Fisher Scientific) prepared from a 1 mM DMSO stock solution and used at 20-200 nM (typically 100 nM) in prewarmed medium. Cells were incubated with the dye for 15-45 min at 37°C, washed with prewarmed medium, and imaged by fluorescence microscopy.

### Measurement of mitochondrial function

Oxygen consumption rate (OCR) and extracellular acidification rate (ECAR) were measured using a Seahorse XFe24 Analyzer (Agilent, Santa Clara, CA, USA) in a 24-well plate format. H1299 and A549 cells (1 × 10^4^) were seeded in XF24 plates at an optimized density and treated with OA-OEt (3-30 μM) for 24 h; the same OA-OEt concentrations were maintained during the assay run. Prior to measurement, cells were incubated in Seahorse assay medium and equilibrated in a non-CO₂ incubator. OCR was recorded at baseline and following sequential injections of oligomycin (2 μM), FCCP (2 μM), and antimycin A (0.5 μM). Mitochondrial respiration parameters, including basal respiration, ATP-linked respiration, maximal respiration, spare respiratory capacity, proton leak, and non-mitochondrial respiration, were calculated using Wave software and normalized to protein content per well.

### Analysis of mitochondrial membrane potential

Mitochondrial membrane potential (ΔΨm) was assessed using the cationic fluorescent probes JC-1 and Rhodamine 123. For JC-1 staining, H1299 and A549 cells were treated with OA-OEt (0, 3, 10, or 30 μM) for 8 h, incubated with JC-1 (5 μg/mL) for 30 min at 37°C in the dark, and then washed with PBS. Fluorescence images were acquired using a Nikon Eclipse Ti fluorescence microscope (Nikon Corporation). The ratio of red (J-aggregates, high ΔΨm) to green (monomers, low ΔΨm) fluorescence was quantified to evaluate changes in ΔΨm. For flow cytometric analysis, cells were treated with OA-OEt (0, 3, 10, or 30 μM) for 8 h and then incubated with JC-1 and Rhodamine 123 (5 μM) for 30 min at 37°C. Fluorescence intensity was measured using a BD Accuri C5 flow cytometer (BD Biosciences). Untreated cells were used as the control group, and data were expressed as a percentage of the control fluorescence.

### Cellular ROS assay

Intracellular reactive oxygen species (ROS) levels were determined using the fluorescent probe 2′,7′-dichlorodihydrofluorescein diacetate (H2DCFDA; Thermo Fisher Scientific). H2DCFDA is a non-fluorescent, cell-permeable compound that is deacetylated by intracellular esterases and subsequently oxidized by ROS to the highly fluorescent 2′,7′-dichlorofluorescein (DCF; excitation ~488 nm, emission ~525-530 nm).H1299 and A549 cells (5 × 10^5 per sample) were treated with OA-OEt (0, 3, 10, or 30 μM) for 1 h at 37°C. For antioxidant rescue experiments, cells were pretreated with N-acetyl-L-cysteine (NAC, 75 mM) for 1 h before exposure to OA-OEt (30 μM, 1 h). After treatment, cells were incubated with 1 μM H2DCFDA at 37°C for 30 min in the dark, washed with PBS, and immediately analyzed by flow cytometry (BD Accuri C5; BD Biosciences). Untreated cells served as the control group. Flow cytometry data were acquired and analyzed using BD Accuri C5 software, and ROS levels were expressed as a percentage of the control mean fluorescence intensity.

### Xenograft assay

All animal experiments were approved by the Institutional Animal Care and Use Committee of Shin Kong Wu Ho Su Memorial Hospital (Taipei, Taiwan; IACUC Approval No. 113NSTCIACUC004) and conducted in accordance with institutional guidelines. Male 4-week-old Nu/Nu mice were purchased from BioLASCO (Taipei, Taiwan) and housed under specific pathogen-free conditions. H1299 cells (2 × 10⁶ cells in 100 μL) were injected subcutaneously into the dorsal flank. When tumors reached ~100 mm³, mice were randomized into control or OA-OEt (50 mg/kg) groups (n = 6 per group) and treated once daily for 24 days by intraperitoneal injection (i.p.) 23, 24. OA-OEt was formulated in 5% DMSO and 95% saline and administered at 100 μL per mouse (corresponding to ~10 mg/mL for a 20 g mouse). Tumor size was measured every 3 days using calipers, and volume was calculated as V = (L × W²)/2, where L is the longest and W the shortest diameter. At the end of the experiment, mice were euthanized by CO₂ inhalation, and tumors were excised for downstream analyses.

### Immunohistochemistry analysis

Paraffin-embedded tumor tissues were sectioned at 4 µm, deparaffinized in xylene, rehydrated through graded ethanol and rinsed in water. After antigen retrieval, immunohistochemistry was performed using the Novolink polymer detection system (Leica, Wetzlar, Germany) according to the manufacturer's instructions. Sections were incubated overnight at 4 °C with primary antibodies against SIRT3, FIS1, DRP1, MFN2, OPA1 and cleaved caspase-3 (1:200), followed by Novolink Polymer for 1 h at room temperature. Signal was developed with 3,3'-diaminobenzidine and counterstained with hematoxylin. Slides were examined by light microscopy, and H-scores were calculated as intensity (0-3) × percentage of positive area (0-100), yielding a total score range of 0-300. H-scores were independently evaluated by three investigators.

### Statistical analysis

Data are presented as mean ± SD. For comparisons among multiple groups, one-way ANOVA was performed followed by Dunnett's post hoc test for comparisons versus the control group, or Tukey's post hoc test for all pairwise multiple comparisons, as appropriate. For comparisons between two groups, an unpaired two-tailed Student's t-test was used. A *p* value < 0.05 was considered statistically significant.

## Results

### Integrated SIRT family analysis identifies SIRT3 as a survival-associated candidate in LUAD

**Figure 1:**
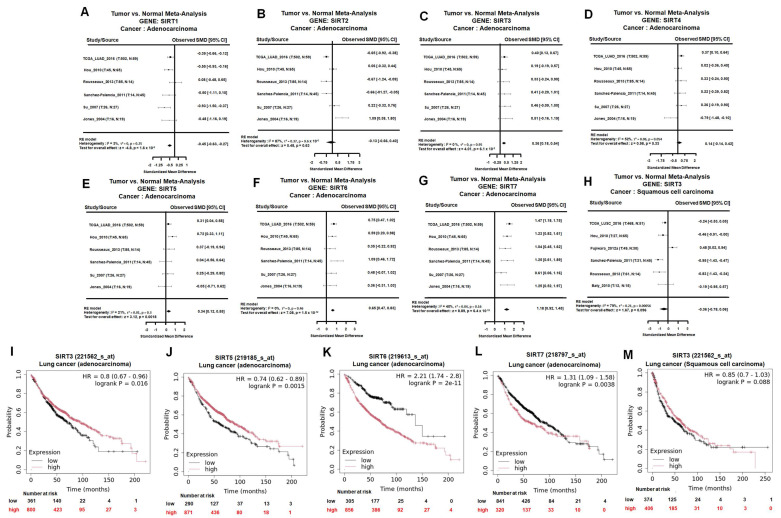
** SIRT family expression and prognostic value in NSCLC.** (A-G) Tumor-versus-normal meta-analysis of SIRT1-SIRT7 mRNA expression in lung adenocarcinoma (LUAD) using the Lung Cancer Explorer platform. Standardized mean differences (SMDs) with 95% confidence intervals were calculated under a random-effects model to compare expression levels between LUAD tissues and corresponding normal lung. (H) Tumor-versus-normal meta-analysis of SIRT3 mRNA expression in lung squamous cell carcinoma (LUSC), showing a nonsignificant trend toward reduced SIRT3 expression in tumors. (I-L) Overall survival analysis of SIRT3, SIRT5, SIRT6, and SIRT7 in LUAD patients. Kaplan-Meier curves depict overall survival stratified by high versus low expression of each SIRT gene based on the Kaplan-Meier Plotter database; log-rank p-values < 0.05 were considered statistically significant. (M) Overall survival analysis of SIRT3 expression in LUSC patients, showing a nonsignificant trend toward better survival in the high-expression group.

To clarify the expression patterns and clinical relevance of SIRT family members in lung cancer, we first performed a tumor-versus-normal meta-analysis across six independent lung adenocarcinoma (LUAD) datasets. Among the seven SIRT family members, SIRT1 was significantly downregulated in LUAD tumor tissues compared with normal lung tissues, whereas SIRT2 and SIRT4 showed no significant differences (Figure 1A-B, D). In contrast, SIRT3, SIRT5, SIRT6, and SIRT7 showed significantly higher mRNA expression in LUAD tissues than in normal lung tissues (Figure 1C, E-G). These findings indicate that individual SIRT family members display distinct transcriptional patterns in LUAD.

We next examined whether the LUAD-associated expression changes of these SIRT family members were related to patient outcomes. Kaplan-Meier survival analysis showed that high SIRT3 and SIRT5 expression was significantly associated with better overall survival in LUAD patients (Figure 1I and L). In contrast, high SIRT6 and SIRT7 expression correlated with poorer overall survival (Figure 1J-K). Thus, although SIRT3, SIRT5, SIRT6, and SIRT7 were all elevated at the mRNA level in LUAD, their prognostic associations were divergent. These results suggest that increased expression of SIRT family members does not uniformly indicate a tumor-promoting role and that SIRT3 may have a survival-beneficial association in LUAD.

We further evaluated SIRT3 expression and prognostic relevance in lung squamous cell carcinoma (LUSC). Meta-analysis of SIRT3 mRNA showed no significant difference between LUSC tumor tissues and normal lung tissues, although a nonsignificant trend toward reduced expression was observed (Figure 1H). Consistently, Kaplan-Meier analysis showed that high SIRT3 expression was not significantly associated with overall survival in LUSC patients (Figure 1M). These findings indicate that the expression pattern and prognostic relevance of SIRT3 may differ between LUAD and LUSC, with a more evident survival-beneficial association in LUAD.

**Figure 2:**
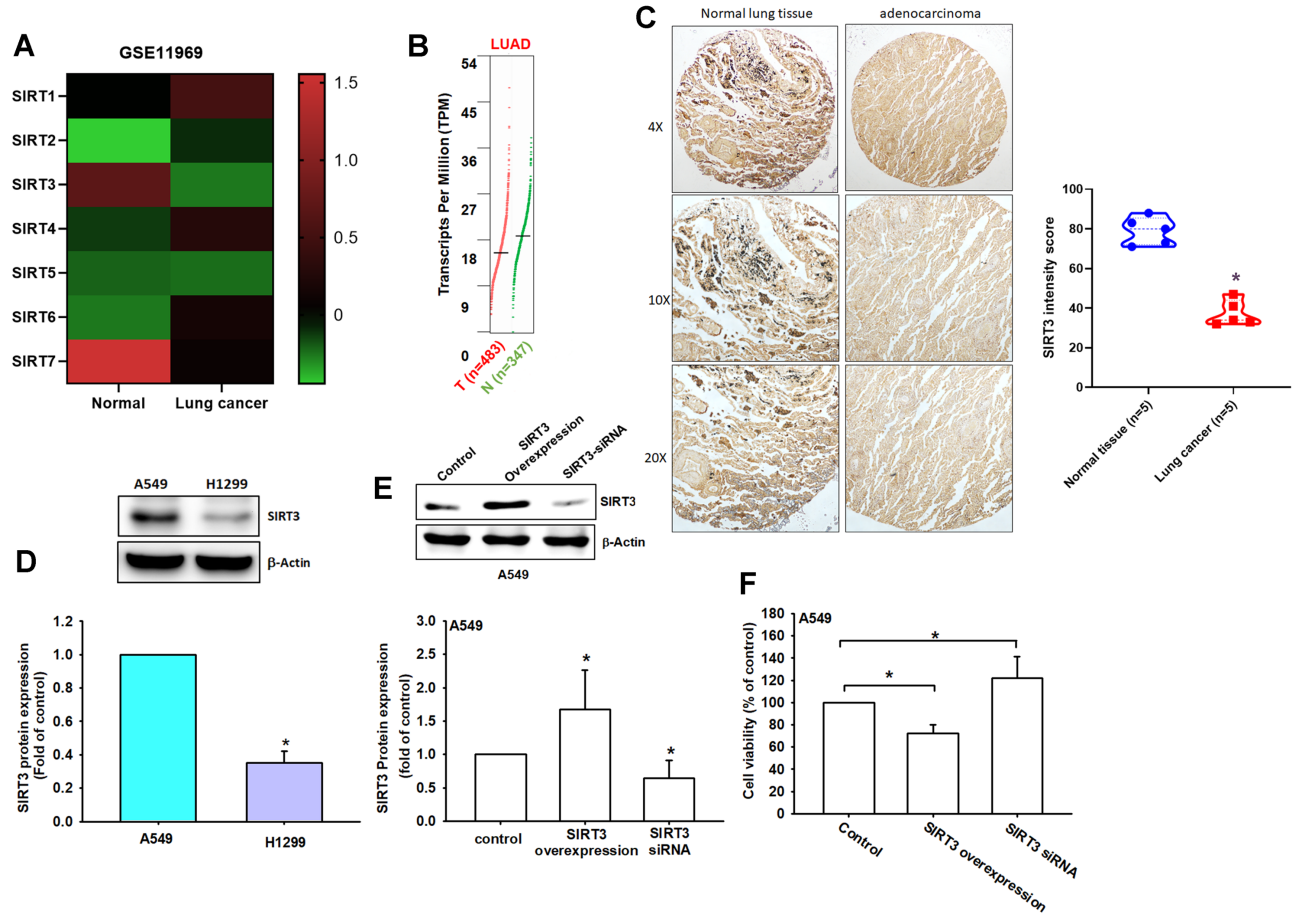
** SIRT3 is downregulated in NSCLC and is associated with cell viability.** (A) Heat map of SIRT family gene expression in normal lung tissues and lung cancer tissues from the GSE11969 dataset (Z-scores). (B) SIRT3 expression in LUAD from GEPIA. (C) Representative IHC images of SIRT3 in normal lung and lung adenocarcinoma tissues (left) and corresponding H-score quantification (right). (D) Basal SIRT3 protein levels in A549 and H1299 cells by immunoblotting (n = 4). (E) Immunoblot confirmation of SIRT3 overexpression or knockdown in A549 cells (n = 4). (F) Cell viability of A549 cells following SIRT3 overexpression or knockdown assessed by CCK-8. Results are shown as means ± SD. *p < 0.05 compared with untreated control.

Based on these results, SIRT3 was selected for further investigation because it showed a favorable prognostic association in LUAD and has an established role in regulating mitochondrial metabolism, redox homeostasis, and cell survival. To further characterize SIRT3 expression, we next performed additional transcriptomic and protein-level validation. In the GSE11969 dataset, SIRT3 and SIRT7 were decreased in lung cancer tissues compared with normal lung tissues (Figure 2A). GEPIA analysis similarly showed lower SIRT3 expression in LUAD tissues relative to normal controls (Figure 2B). These results suggest that SIRT3 mRNA expression may vary across transcriptomic cohorts, highlighting the need for protein-level validation.

Importantly, immunohistochemical analysis of clinical specimens showed markedly weaker SIRT3 staining in lung adenocarcinoma tissues than in normal lung tissues, with significantly lower H-scores in tumor samples (normal: 80.3 ± 12.1 vs. tumor: 45.2 ± 18.7; Figure 2C). Western blot analysis further revealed differential SIRT3 protein expression in NSCLC cell lines, with H1299 cells displaying lower SIRT3 levels than A549 cells (Figure 2D). These findings support reduced SIRT3 protein expression in lung cancer tissues and suggest that SIRT3 expression may be regulated differently at the transcript and protein levels.

To determine whether SIRT3 has functional relevance in lung cancer cells, we next modulated SIRT3 expression in A549 cells. SIRT3 overexpression significantly reduced cell viability, whereas SIRT3 knockdown increased cell proliferation (Figure 2E-F). These gain- and loss-of-function results indicate that SIRT3 negatively regulates NSCLC cell growth. Collectively, these findings support SIRT3 as a prognostically associated and functionally relevant candidate for subsequent mechanistic investigation in NSCLC.

### Structure-based analysis of OA-OEt as a candidate SIRT3 modulator

**Figure 3:**
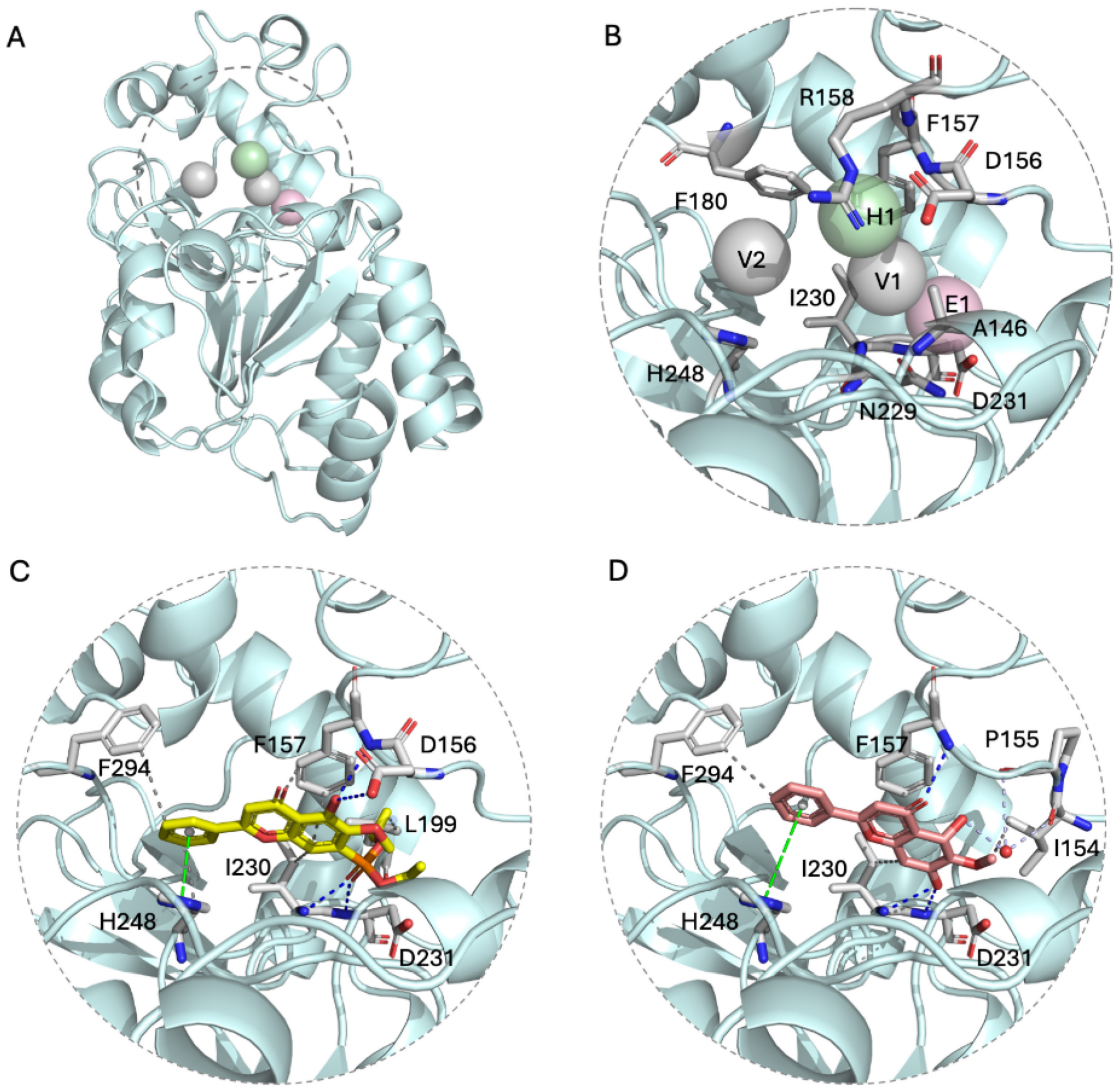
** Structure-based analysis indicated OA-OEt as a candidate SIRT3 modulator.** (A) SIRT3 structure (PDB ID: 4JSR) with binding pocket (shown by dashed circle) and interaction anchors (shown by spheres). (B) Binding pocket map showing E1 (electrostatic), H1 (hydrogen bonding), and V1/V2 (van der Waals) interaction anchors and key residues contributing to binding preferences. (C-D) Predicted binding modes of OA-OEt (C) and OA (D) in the SIRT3 pocket. Gray dotted line: hydrophobic interactions; Blue dotted line: hydrogen bonds; Green dotted line: π-stacking; Lavender dotted line: water bridges.

To evaluate potential SIRT3 modulators, structure-based analyses were performed using the SIRT3 crystal structure (PDB: 4JSR). The binding pocket was characterized by four key interaction anchors: E1 (electrostatic contacts with Asp231), H1 (hydrogen bonds with Asp156, Phe157, and Arg158) and V1/V2 (van der Waals interactions with Ala146, Phe157, Asn229, Phe180, Ile230, and His248) (Figure 3A-B). The E1 anchor, centered on the negatively charged side chain of Asp231, preferentially accommodates phosphate groups. The H1 anchor, situated within a loop critical for NAD⁺ binding, comprises negatively charged Asp156, hydrophobic Phe157, and positively charged Arg158. The V1 and V2 anchors consist of hydrophobic residues (Ala146, Phe157, Phe180, and Ile230) and polar Asn229; notably, His248 and Asn229 constitute part of the catalytic site, favoring aromatic moieties (Figure S1). Molecular docking via iGEMDOCK, integrated with SiMMap analysis, revealed favorable binding modes for oroxylin A (OA) and its derivative, oroxylin A phosphate diethyl ester (OA-OEt). Intermolecular interactions between SIRT3 and OA/OA-OEt were identified by PLIP (Figure 3C-D). Specifically, the docked SIRT3-OA-OEt complex involved hydrophobic interactions (Phe157, Leu199, Ile230, His248, and Phe294), hydrogen bonds (Asp156, Phe157, Ile230, and Asp231), and π-stacking (His248). Compared to OA, OA-OEt exhibited a higher frequency of hydrophobic interactions, whereas the hydrogen bond with Asp156 was absent in the OA-bound complex. Conversely, two water-bridge interactions (Ile154 and Pro155) were uniquely observed in the OA binding mode. Details of the interaction forces are shown in Figure S2.

OA-OEt, synthesized by substituting the hydroxyl group of OA with a phosphodiethyl ester, demonstrated efficient binding to SIRT3. This improvement was attributed to strengthened electrostatic interactions with the H1 anchor and augmented van der Waals interactions. Collectively, these *in silico* analyses prioritize OA-OEt as a promising SIRT3 modulator with an optimized interaction profile for subsequent experimental validation.

### OA-OEt potently activates SIRT3 and inhibits lung cancer cell growth

**Figure 4:**
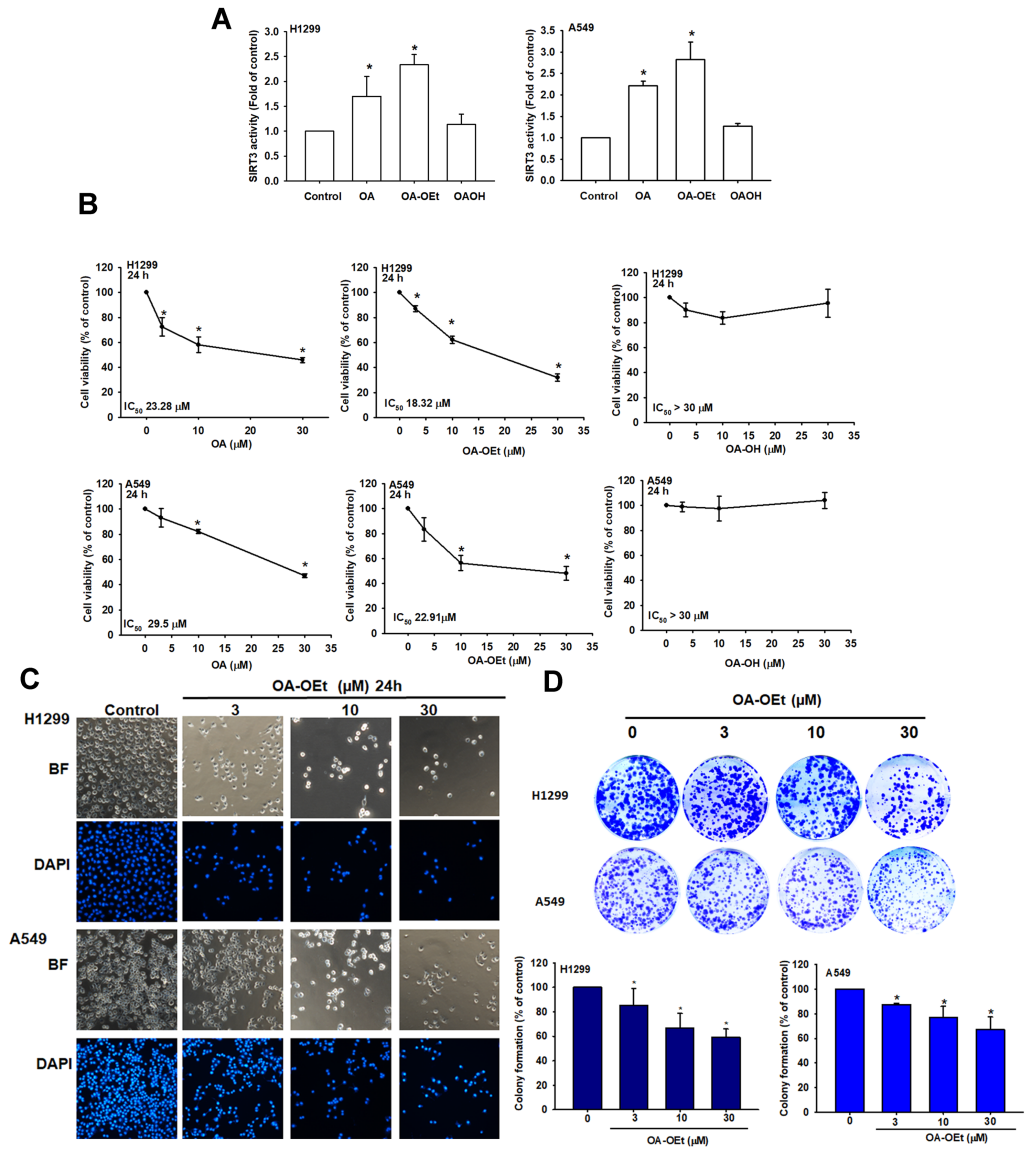
** OA-OEt increases SIRT3 activity and suppresses proliferation of NSCLC cells.** (A) SIRT3 activity in H1299 and A549 cells after treatment with OA, OA-OEt, or OA-OH (n = 4). (B) Cell viability of H1299 and A549 cells treated with OA, OA-OEt, or OA-OH (3-30 μM) for 24 h assessed by CCK-8 (n = 4). (C) Representative bright-field images and DAPI staining showing morphological changes and chromatin condensation after OA-OEt treatment (3-30 μM, 24 h). (D) Colony formation after short OA-OEt exposure (3-30 μM, 6 h) followed by culture in drug-free medium for 14 days (n = 4). Results are shown as means ± SD. *p < 0.05 compared with untreated control. **p < 0.01 compared with untreated control.

To assess the efficacy of the identified compounds, we first measured SIRT3 activity in lung cancer cells treated with OA, OA-OEt, or OA-OH. SIRT3 activity assays confirmed that both OA and OA-OEt significantly increased SIRT3 activity, with OA-OEt showing 2-fold greater activation than OA in H1299 and A549 cell lines (Figure 4A). Cell viability assessments revealed that OA-OEt exhibited superior antiproliferative effects with IC₅₀ values of 18.32 ± 0.8 μM in H1299 cells and 22.91 ± 0.8 μM in A549 cells, compared to 23.28 ± 0.7 μM and 29.5 ± 1.1 μM for OA, respectively (Figure 4B). Morphological analysis revealed dose-dependent cellular changes, including membrane blebbing and nuclear condensation characteristics of apoptosis (Figure 4C). DAPI staining confirmed chromatin condensation and nuclear fragmentation (Figure 4C). In colony-formation assays, even low concentrations of OA-OEt (3 µM) markedly impaired long-term proliferative capacity, reducing colony formation by more than 80% (Figure 4D). Together, these findings underscore the potent anticancer potential of OA-OEt and motivated subsequent studies to delineate the mechanisms by which OA-OEt induces cell death in lung cancer cells.

### OA-OEt induces G2/M cell cycle arrest and apoptosis

**Figure 5:**
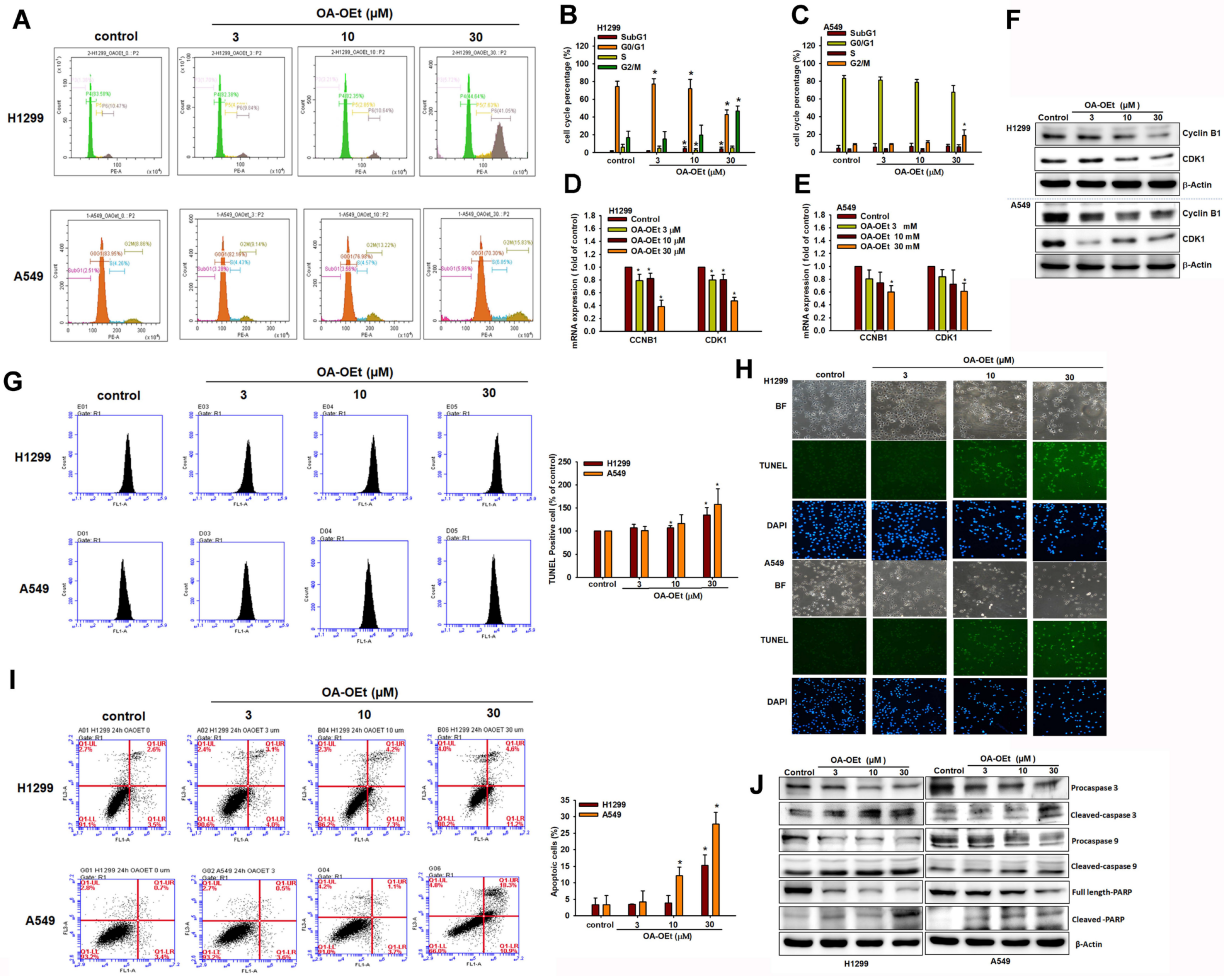
** OA-OEt induces G2/M arrest and apoptosis in NSCLC cells.** (A-C) Cell-cycle distribution of H1299 and A549 cells treated with OA-OEt (3-30 μM, 24 h) and stained with PI for flow cytometry (n = 4). (D-E) CCNB1 and CDK1 mRNA levels after OA-OEt treatment (3-30 μM, 24 h) measured by qPCR. (F) CDK1 and cyclin B1 protein levels after OA-OEt treatment (3-30 μM, 6 h) by immunoblotting (n = 4). (G-H) TUNEL assay and DAPI staining showing DNA fragmentation and nuclear changes after OA-OEt treatment (3-30 μM, 24 h). (I) Annexin V/PI staining after OA-OEt treatment (3-30 μM, 8 h) analyzed by flow cytometry (n = 4). (J) Cleaved caspase-3/9 and PARP after OA-OEt treatment (3-30 μM, 8 h) by immunoblotting (n = 4). Untreated cells were used as controls. Results are shown as means ± SD. *p < 0.05 compared with untreated control.

OA and its derivatives have been reported to exert significant anti-cancer effects in a variety of cancer cells by inducing apoptosis and inhibiting cell proliferation 12. To determine whether OA-OEt-mediated growth inhibition is associated with alterations in cell-cycle progression in NSCLC, we analyzed DNA content by flow cytometry. OA-OEt treatment led to a dose-dependent accumulation of cells in G2/M phase, accompanied by a concomitant reduction in the G0/G1 population (Figure 5A-C). This shift was associated with a marked decrease in the expression of the G2/M regulators CDK1 and cyclin B1 at both the mRNA and protein levels (Figure 5D-F). To further investigate whether OA-OEt induces cell death, we examined apoptotic hallmarks. TUNEL assays revealed increased DNA fragmentation in OA-OEt-treated cells (Figure 5G-H). Consistently, annexin V/propidium iodide staining revealed dose-dependent increases in both early and late apoptotic cell populations (Figure 5I). Western blot analysis confirmed activation of the intrinsic apoptotic pathway, with increased levels of cleaved caspase-3, caspase-9, and PARP following OA-OEt treatment (Figure 5J). Taken together, these results indicate that the anti-cancer activity of OA-OEt in lung cancer cells is mediated, at least in part, through induction of G2/M cell-cycle arrest and apoptosis.

### SIRT3-associated genes are enriched in mitochondrial and cell cycle pathways

**Figure 6:**
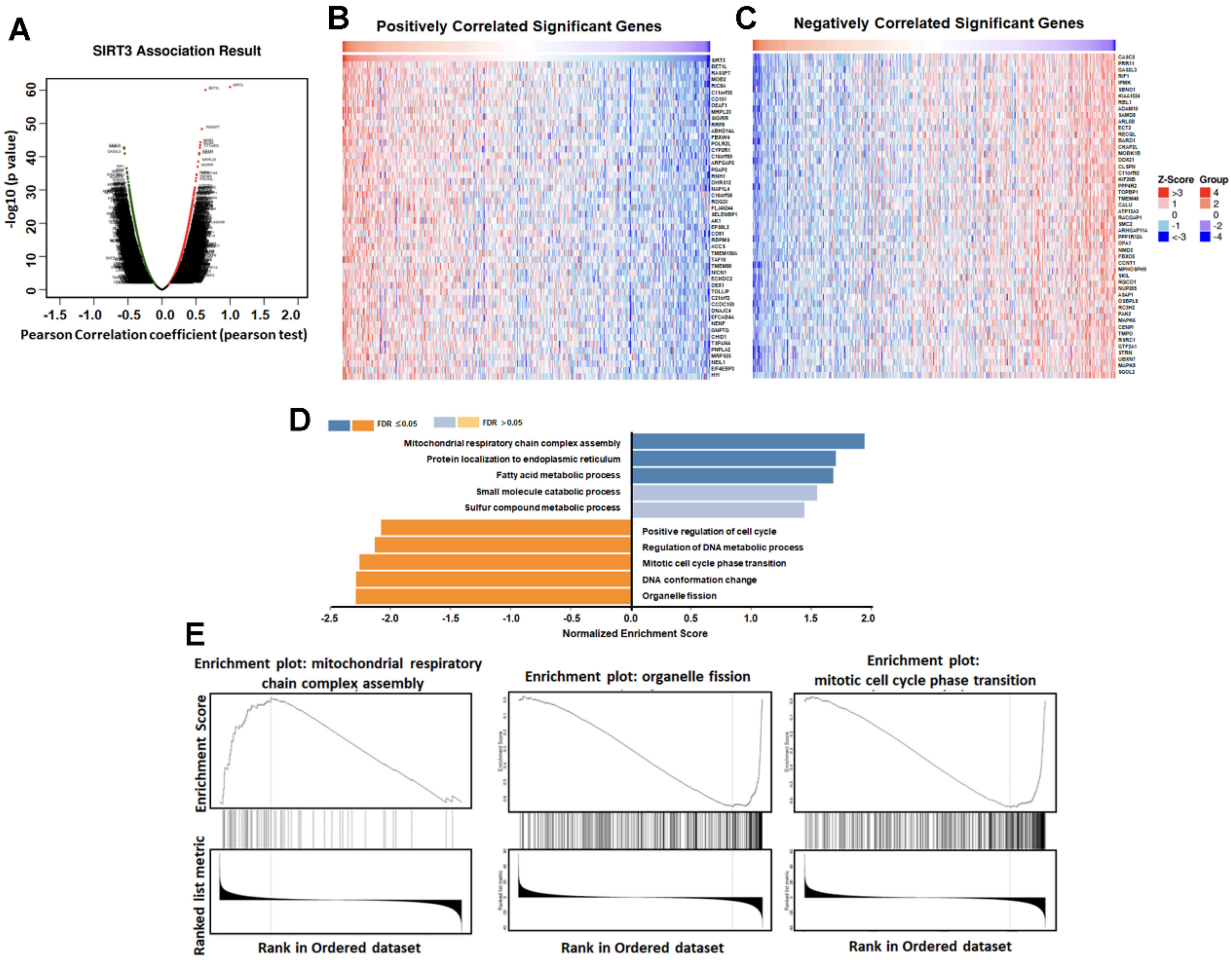
** SIRT3-associated genes are enriched in mitochondrial metabolism and cell-cycle pathways.** (A) Volcano plot of genes correlated with SIRT3 in LUAD (LinkedOmics). (B) Top 50 genes positively correlated with SIRT3. (C) Top 50 genes negatively correlated with SIRT3. (D) GO enrichment analysis of SIRT3-associated genes. (E) GSEA of SIRT3-associated gene sets.

To gain further insight into the functional roles of SIRT3, we utilized the LinkedOmics database to identify the top 50 genes positively or negatively correlated with SIRT3 expression in LUAD. GO and GSEA analyses indicated that genes positively correlated with SIRT3 were enriched in mitochondrial energy and fatty acid metabolism, whereas negatively correlated genes were enriched in mitotic cell-cycle regulation and organelle fission (Figure 6A-E). These findings support a model in which SIRT3 couples mitochondrial metabolic programs to restrain proliferative signaling and provide a rationale for exploring SIRT3 activation as a therapeutic strategy in lung cancer.

### OA-OEt disrupts mitochondrial homeostasis and induces oxidative stress

**Figure 7:**
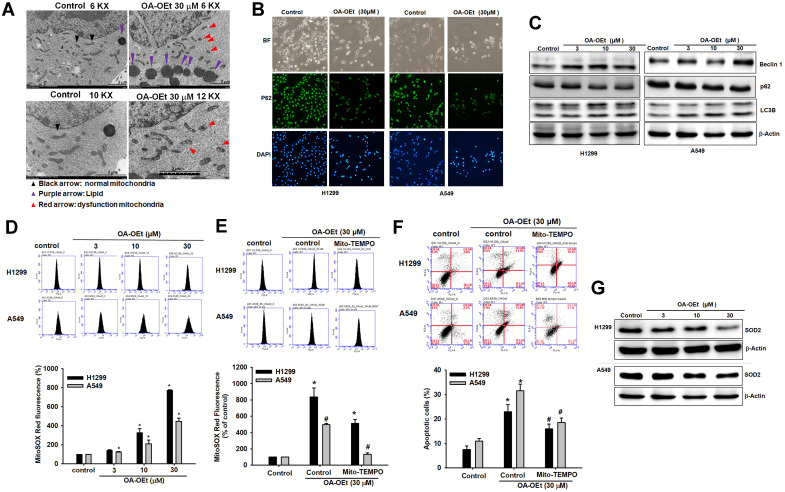
** OA-OEt alters mitochondrial morphology, autophagy-related markers, and mitochondrial superoxide in NSCLC cells.** (A) TEM images of H1299 cells treated with OA-OEt (30 μM, 8 h). Lipid droplets (purple arrows); representative normal mitochondria (black arrows) and abnormal mitochondria (red arrows). Scale bar = 2 μm. (B) Immunofluorescence staining of p62 after OA-OEt treatment (30 μM, 8 h). (C) Immunoblotting of Beclin-1, p62, and LC3B after OA-OEt treatment (3-30 μM, 8 h) (n = 4). (D) MitoSOX staining after OA-OEt treatment (3-30 μM, 2 h) followed by incubation with MitoSOX for 10 min and flow cytometry (n = 4). (E) Cells pretreated with Mito-TEMPO (1 h) and then treated with OA-OEt (3-30 μM, 2 h); mitochondrial superoxide measured by MitoSOX/flow cytometry (n = 4). (F) Cells pretreated with Mito-TEMPO (1 h) and then treated with OA-OEt (3-30 μM, 8 h); apoptosis assessed by Annexin V/PI flow cytometry (n = 4). (G) SOD2 protein levels after OA-OEt treatment (3-30 μM, 24 h) by immunoblotting (n = 4). Untreated cells were used as controls. Results are shown as means ± SD. *p < 0.05 compared with untreated control. #*p* < 0.05 compared with OA-OEt alone.

SIRT3 has been implicated in cancer progression through its regulation of mitochondrial function and metabolism 4-6. Dysregulation of mitochondrial fission and fusion dynamics contributes to tumor progression and metastasis, with excessive fission driving metabolic reprogramming, proliferation and resistance to apoptosis, and also promoting mitochondrial ROS (mtROS) production and mitophagy 7-10, 25. To determine whether OA-OEt impacts mitochondrial homeostasis, we performed transmission electron microscopy (TEM) on H1299 cells treated with OA-OEt (30 µM). TEM analysis revealed increased intracellular lipid droplet accumulation and a shift in mitochondrial morphology from elongated to fragmented structures (Figure 7A). Immunofluorescence staining showed a reduction in p62 expression, suggestive of increased autophagy-related signaling (Figure 7B), which was corroborated by western blot analysis demonstrating increased LC3B and Beclin-1 and decreased p62 levels following OA-OEt treatment (Figure 7C). Flow cytometry using MitoSOX Red staining revealed a rapid and robust increase in mitochondrial superoxide production within 2 h of OA-OEt exposure, reaching approximately an 8-fold elevation in H1299 cells and a 5-fold elevation in A549 cells at 30 µM (Figure 7D). Pretreatment with the mitochondrial-targeted antioxidant Mito-TEMPO significantly attenuated OA-OEt-induced ROS production and partially rescued apoptosis, supporting a contributory role of oxidative stress in OA-OEt cytotoxicity (Figure 7E-F). Furthermore, OA-OEt downregulated SOD2 expression, further weakening mitochondrial antioxidant defenses and exacerbating oxidative stress (Figure 7G). To functionally validate the link between SIRT3 activation and mitochondrial regulation, we then assessed mitochondrial membrane potential (ΔΨm) in H1299 and A549 cells using JC-1 and Rhodamine 123 staining. Fluorescence microscopy showed that OA-OEt (3-30 μM, 8 h) increased JC-1 red fluorescence compared with controls (Figure S3A), and quantitative analysis revealed a significant, dose-dependent elevation of the JC-1 red/green fluorescence ratio, indicative of increased ΔΨm consistent with mitochondrial hyperpolarization under stress conditions (Figure S3B). In line with these findings, flow cytometric analysis of Rhodamine 123 staining demonstrated a dose-dependent increase in fluorescence intensity after OA-OEt treatment in both H1299 and A549 cells (Figure S3C-D). Together with OA-OEt-induced mitochondrial fragmentation, mtROS accumulation, and impaired oxidative phosphorylation described above, these data suggest that OA-OEt elicits a transient enhancement of ΔΨm as part of a dynamic mitochondrial stress response that precedes global mitochondrial dysfunction in NSCLC cells.

Finally, we examined whether OA-OEt also modulates total intracellular ROS levels. H2DCFDA-based flow cytometry showed that OA-OEt treatment (3-30 μM, 1 h) induced a marked, dose-dependent increase in DCF fluorescence in both H1299 and A549 cells compared with untreated controls (Figure S4A-B). At 30 μM, OA-OEt elevated intracellular ROS to approximately 10-fold of control in H1299 cells and 3-fold of control in A549 cells. Importantly, pretreatment with the antioxidant N-acetyl-L-cysteine (NAC) dramatically reduced OA-OEt-induced DCF fluorescence in both cell lines, restoring ROS levels toward near-baseline values (Figure S4C-D). Collectively, these findings indicate that the anticancer effects of OA-OEt are tightly linked to the induction of mitochondrial dysfunction—encompassing altered mitochondrial morphology, lipid accumulation, autophagy activation, excessive superoxide generation, and global oxidative stress—which ultimately culminates in apoptotic cell death in lung cancer cells.

### OA-OEt promotes mitochondrial fission and impairs bioenergetic function

**Figure 8:**
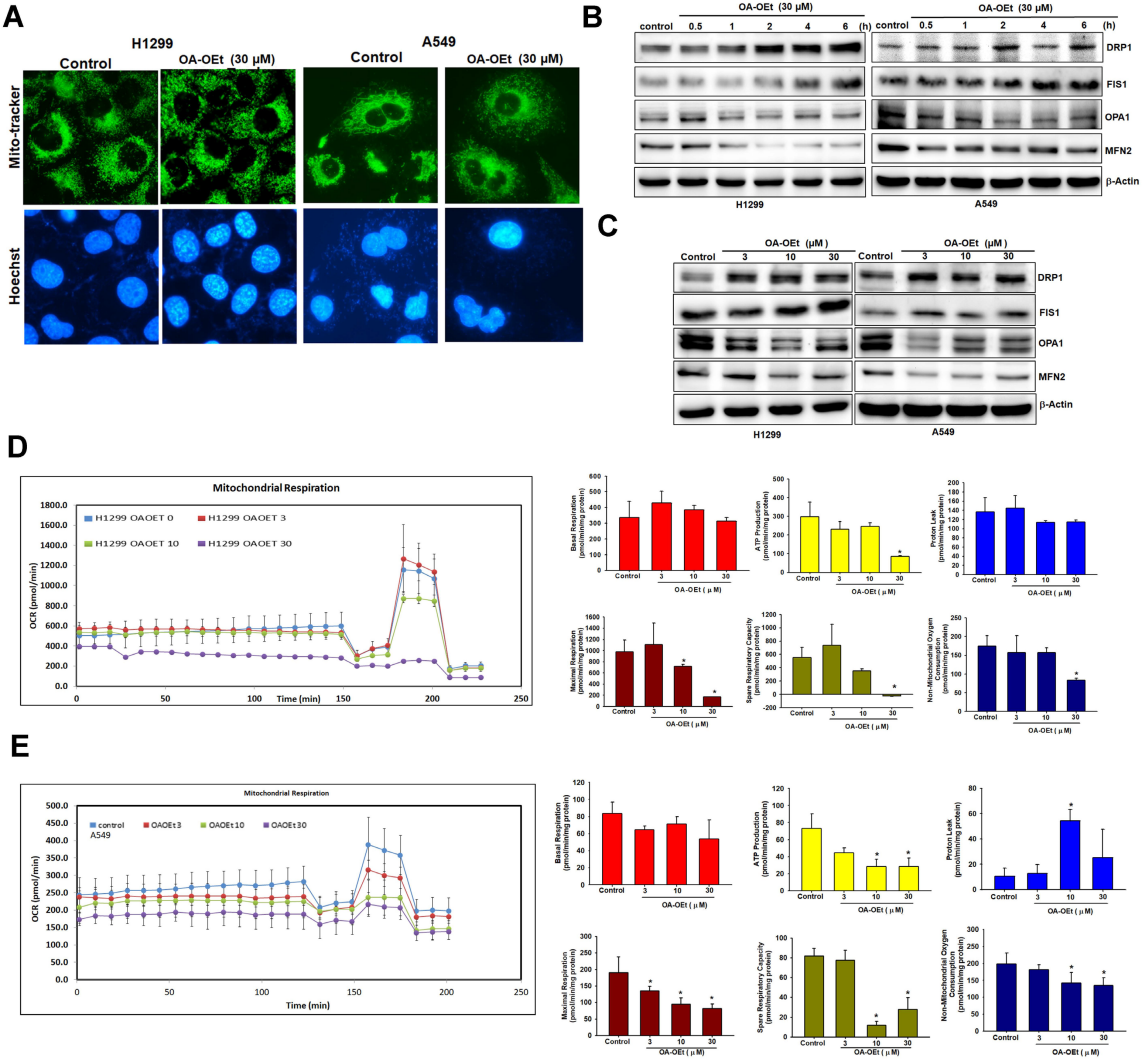
** OA-OEt promotes mitochondrial fission and impairs mitochondrial respiration in NSCLC cells.** (A) Mitochondrial morphology in H1299 cells after OA-OEt treatment (30 μM, 2 h) stained with MitoTracker™ (mitochondria) and Hoechst 33342 (nuclei). (B) Time-course immunoblotting of DRP1, FIS1, OPA1, and MFN2 after OA-OEt treatment (30 μM, 0.5-6 h) in H1299 and A549 cells (n = 4). (C) Dose-response immunoblotting of DRP1, FIS1, OPA1, and MFN2 after OA-OEt treatment (3-30 μM, 2 h) (n = 4). (D-E) Seahorse analysis of OCR in H1299 and A549 cells treated with OA-OEt (3-30 μM) for 24 h, with the same concentrations maintained during the assay. OCR was measured at baseline and after sequential injections of oligomycin (2 μM), FCCP (2 μM), and antimycin A (0.5 μM) (n = 4). Basal respiration, maximal respiration, ATP-linked respiration, spare respiratory capacity, non-mitochondrial respiration, and proton leak are shown. Untreated cells were used as controls. Results are shown as means ± SD. *p < 0.05 compared with untreated control. **p < 0.01 compared with untreated control.

To assess the effects of OA-OEt on mitochondrial dynamics, we stained OA-OEt-treated NSCLC cells with MitoTracker and examined mitochondrial morphology by fluorescence microscopy. MitoTracker staining revealed a marked shift from elongated, interconnected mitochondrial networks to fragmented, punctate structures following OA-OEt treatment (Figure 8A). Western blot analysis further demonstrated time- and dose-dependent increases in the fission markers DRP1 and FIS1, accompanied by concomitant decreases in the fusion markers OPA1 and MFN2 (Figure 8B-C). Mitochondrial respiration was next evaluated using Seahorse metabolic flux analysis. In H1299 cells, OA-OEt induced dose-dependent reductions in ATP production (approximately 300 to 100), maximal respiration (approximately 1000 to 200), and spare respiratory capacity (approximately 570 to -20). Similarly, in A549 cells, OA-OEt treatment at 30 µM significantly decreased ATP production (approximately 75 to 25), maximal respiration (approximately 190 to 90), and spare respiratory capacity (approximately 80 to 25; Figure 8D-E). Together, these data indicate that OA-OEt induces severe mitochondrial dysfunction and bioenergetic collapse in NSCLC cells.

### OA-OEt inhibits tumor growth *in vivo* and modulates mitochondrial dynamics

**Figure 9:**
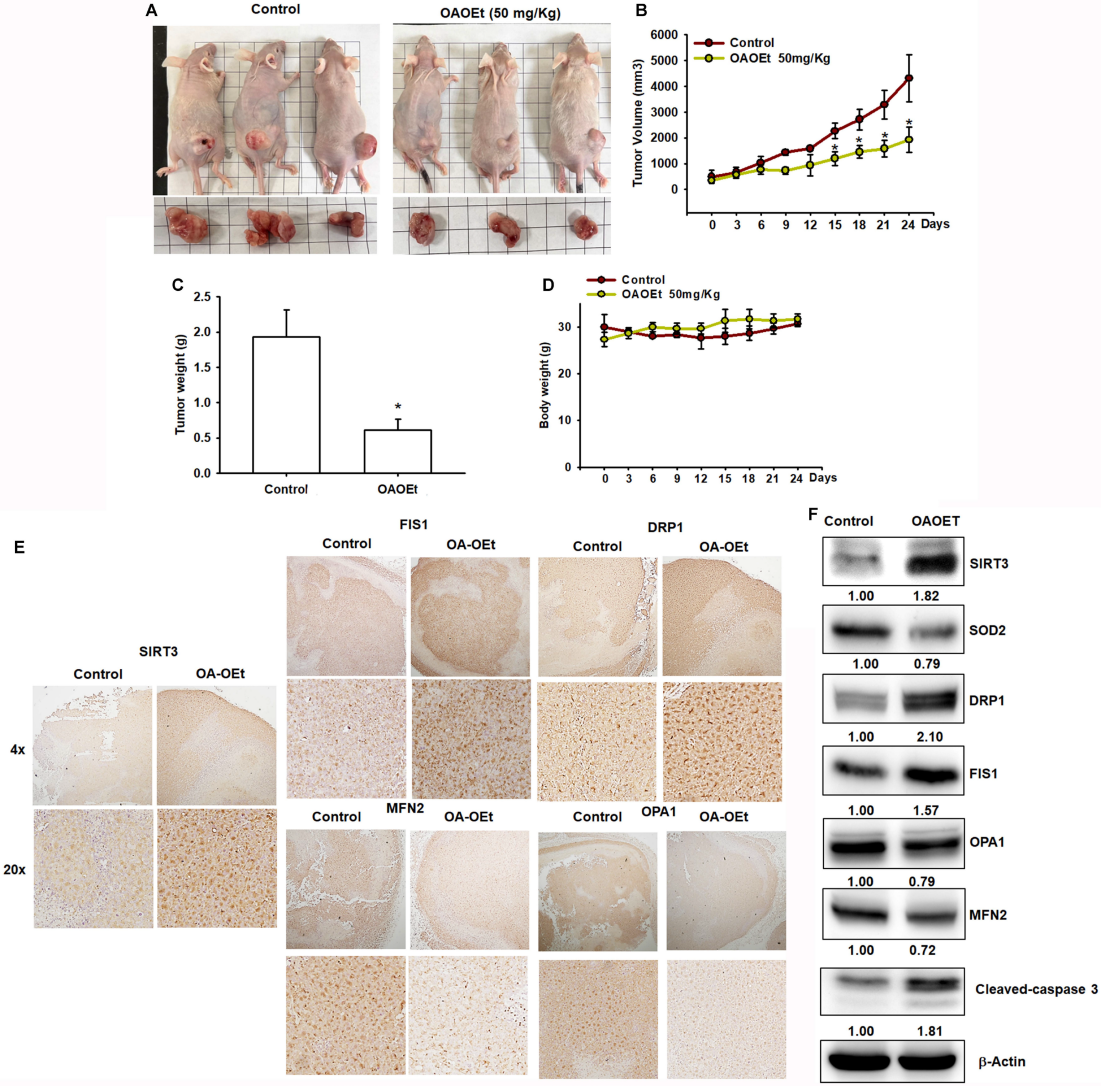
** OA-OEt suppresses xenograft growth and is associated with altered mitochondrial dynamics and apoptosis *in vivo*.** (A) Representative images of mice and excised tumors. (B-D) Tumor volume over time, final tumor weight at endpoint, and body weight in control- and OA-OEt-treated groups (n = 6). (E) IHC staining of SIRT3, DRP1, FIS1, MFN2, and OPA1 in xenograft tumors from control and OA-OEt-treated mice. (F) Immunoblotting of SIRT3, SOD2, DRP1, FIS1, MFN2, OPA1, and cleaved caspase-3 in tumor lysates (n = 4). Results are shown as means ± SD. *p < 0.05 compared with control group.

To evaluate the therapeutic efficacy of OA-OEt *in vivo*, we established H1299 xenografts in nude mice. Daily administration of OA-OEt (50 mg/kg) significantly suppressed tumor growth, with tumor volumes reaching approximately 2,000 ± 300 mm³ in the OA-OEt group compared to 4,500 ± 500 mm³ in controls by day 24, representing a 56% reduction in tumor growth (Figure 9A-C). Consistently, final tumor weights were significantly decreased in the OA-OEt-treated group (0.65 ± 0.15 g) compared to controls (1.95 ± 0.35 g, Figure 9C). Importantly, OA-OEt treatment did not significantly affect body weight throughout the study period, indicating acceptable tolerability (Figure 9D). Immunohistochemical analysis of xenograft tumor tissues revealed increased SIRT3 expression in OA-OEt-treated tumors, along with elevated levels of mitochondrial fission markers (DRP1, FIS1) and reduced fusion markers (MFN2, OPA1) (Figure 9E). These changes were corroborated by western blot analysis, which showed increased protein levels of SIRT3 and the mitochondrial fission markers DRP1 and FIS1, together with decreased levels of SOD2 and the fusion markers OPA1 and MFN2 in OA-OEt-treated xenograft tumors. Western blotting also revealed elevated cleaved caspase-3, consistent with increased apoptosis (Figure 9F). Notably, reduced SOD2 in xenograft tumors was consistent with the OA-OEt-induced oxidative stress phenotype observed in NSCLC cells *in vitro*. Collectively, OA-OEt suppressed NSCLC tumor growth and was associated with altered mitochondrial dynamics markers and enhanced apoptosis in xenograft tumors.

## Discussion

This study supports SIRT3 as a functionally relevant mitochondrial regulator and potential therapeutic vulnerability in NSCLC and illustrates how structure-based approaches can be used to prioritize small-molecule SIRT3 modulators for experimental validation. Our integrative analyses showed that SIRT3 displays cohort-dependent transcriptomic variation in lung cancer. Although SIRT3 mRNA was modestly elevated in the integrated LUAD meta-analysis, higher SIRT3 expression was associated with better overall survival in LUAD patients. Additional transcriptomic analyses using GSE11969 and GEPIA, together with protein-level validation by immunohistochemistry, further supported reduced SIRT3 expression in lung cancer specimens. These findings suggest that SIRT3 expression and prognostic relevance in lung cancer may be influenced by tumor subtype, dataset composition, and regulatory differences between transcript and protein levels. These observations are notable given the context-dependent dual roles of SIRT3 reported across malignancies.

SIRT3 is a mitochondrial NAD⁺-dependent deacetylase that regulates mitochondrial metabolism, redox balance, and cell survival. While SIRT3 can support tumor progression in certain settings, substantial evidence indicates tumor-suppressive functions in others by limiting oxidative stress and metabolic reprogramming 4-6. For example, SIRT3 can activate antioxidant programs such as SOD2 and restrain ROS-driven signaling, and loss of SIRT3 has been linked to increased glycolysis and angiogenic signaling in some cancers 6, 26. Conversely, SIRT3 may promote tumor cell fitness by maintaining mitochondrial function and metabolic flexibility in other contexts 5, 6. In our NSCLC analyses, the favorable survival association of SIRT3 in LUAD, reduced SIRT3 protein expression in lung cancer specimens, and the growth-suppressive effect of SIRT3 overexpression collectively support a tumor-suppressive role for SIRT3 in NSCLC cells.

To pharmacologically engage SIRT3, we performed structure-based virtual screening and prioritized OA-OEt, a phosphodiethyl ester derivative of oroxylin A, for experimental testing. OA-OEt increased SIRT3 activity in cell lysates and showed stronger antiproliferative effects than the parent compound in NSCLC cells 11-15. OA-OEt induced G2/M arrest accompanied by reduced cyclin B1 and CDK1 expression and activated intrinsic apoptosis, as indicated by caspase-3/9 and PARP cleavage together with TUNEL and Annexin V assays.

Mitochondrial permeability transition pores (mPTP) are nonselective channels in the inner mitochondrial membrane, and sustained mPTP opening can disrupt mitochondrial membrane potential, alter ROS production, and promote downstream events such as mitochondrial fission and autophagy, culminating in apoptosis 27. mPTP-related mitochondrial stress has also been linked to activation of fission machinery (e.g., DRP1) and to selective clearance of damaged mitochondria via mitophagy in some settings 28, 29. Although mPTP opening and mitophagy were not directly measured here, these pathways provide a useful framework for interpreting the mitochondrial stress phenotypes observed after OA-OEt treatment. Mitochondrial fusion and fission are closely coupled to changes in ΔΨm, and individual daughter mitochondria can display divergent fates after fission, with some units undergoing sustained depolarization whereas others become relatively hyperpolarized. In our system, OA-OEt not only promoted mitochondrial fragmentation and shifted fission-fusion markers toward fission but also induced a dose-dependent increase in the JC-1 red/green ratio and Rhodamine 123 fluorescence, consistent with transient mitochondrial hyperpolarization under stress conditions. Such hyperpolarization has been proposed to represent a compensatory response during mitochondrial quality control, temporarily maintaining ATP production in a subset of mitochondria while persistently depolarized organelles are targeted for autophagic removal 30, 31. Taken together, our data are consistent with a model in which OA-OEt induces excessive mitochondrial fission, mtROS accumulation, and suppression of SOD2 that progressively overwhelm these adaptive responses, thereby contributing to global mitochondrial dysfunction, bioenergetic collapse, and apoptosis in NSCLC cells.

Our data indicate that OA-OEt perturbs mitochondrial homeostasis and bioenergetics. TEM and fluorescence microscopy revealed mitochondrial fragmentation and lipid accumulation after OA-OEt exposure. OA-OEt increased mitochondrial superoxide and reduced SOD2 expression, and it altered autophagy-related markers, including LC3B and Beclin-1 upregulation with p62 reduction. Importantly, the mitochondrial ROS scavenger Mito-TEMPO attenuated superoxide accumulation and partially rescued apoptosis, supporting a contributory role of oxidative stress in OA-OEt cytotoxicity 27, 32. OA-OEt also shifted mitochondrial dynamics markers toward fission, as shown by increased DRP1 and FIS1 and decreased OPA1 and MFN2, and impaired oxidative phosphorylation, with reduced oxygen consumption rate, ATP-linked respiration, and spare respiratory capacity in Seahorse assays. Together, these findings support a model in which OA-OEt exposes mitochondrial and redox vulnerabilities in NSCLC cells through combined oxidative stress and mitochondrial remodeling 7-10, 28.

*In vivo*, OA-OEt suppressed H1299 xenograft growth and reduced final tumor weight without overt changes in body weight during treatment. Tumor tissues showed increased SIRT3 and cleaved caspase-3, together with marker changes consistent with increased fission, including DRP1 and FIS1, decreased fusion, including OPA1 and MFN2, and reduced antioxidant defense, as indicated by decreased SOD2. These *in vivo* marker changes are consistent with our *in vitro* observations, but direct target engagement and more comprehensive tolerability assessments (e.g., serum chemistry and organ histopathology) will strengthen translational interpretation.

From a translational perspective, our findings support SIRT3 as a potentially druggable mitochondrial vulnerability in NSCLC. This conclusion is supported by the favorable survival association of SIRT3 in LUAD, reduced SIRT3 protein expression in lung cancer specimens, the growth-suppressive effect of SIRT3 overexpression, and the ability of OA-OEt to activate SIRT3 while suppressing NSCLC growth. Our data further suggest that OA-OEt, a stabilized derivative of oroxylin A, functions as a pharmacologic SIRT3 activator that exposes mitochondrial and redox vulnerabilities by promoting mitochondrial fragmentation, oxidative stress, and bioenergetic collapse. This mechanism is distinct from current oncogene-targeted therapies, such as EGFR, ALK, and ROS1 inhibitors, which mainly inhibit upstream signaling and are often limited by acquired resistance 5, 6. In principle, targeting SIRT3 with OA-OEt could complement these agents by acting on mitochondrial machinery that supports metabolic plasticity and therapy tolerance. SIRT3-directed modulation of mitochondrial stress and ROS-mediated cell death may also be exploitable in rational combinations with chemotherapy, radiotherapy, or immunotherapy 26. However, further work is needed to define pharmacokinetics, safety, direct target engagement, and efficacy in combination regimens in appropriate NSCLC models before clinical translation can be considered.

Collectively, this study provides several biologically and translationally relevant insights. First, SIRT3 emerges as a prognostically and functionally relevant mitochondrial regulator in NSCLC, with a favorable survival association in LUAD and growth-suppressive activity in lung cancer cells. Second, OA-OEt represents a SIRT3-activating lead compound with *in vitro* and *in vivo* activity that warrants further optimization, including structure-activity relationship studies, pharmacokinetic profiling, and direct target engagement assays. Third, targeting mitochondrial stress and redox homeostasis through SIRT3 modulation may offer a mechanistically distinct strategy for NSCLC, including settings where current targeted and immune therapies provide limited durable benefit.

## Conclusion

In summary, our findings support SIRT3 as a functionally tumor-suppressive mitochondrial regulator with a favorable prognostic association in LUAD. OA-OEt activates SIRT3 and suppresses NSCLC growth *in vitro* and *in vivo*, accompanied by disrupted mitochondrial homeostasis, oxidative stress, bioenergetic dysfunction, and apoptosis. These findings support OA-OEt as a promising SIRT3-activating lead compound and highlight SIRT3-associated mitochondrial stress as a potential therapeutic vulnerability in NSCLC.

## Supplementary Material

Supplementary figures and tables.

## Data Availability

The datasets used and/or analyzed during the current study are available from the corresponding author on reasonable request.

## Abbreviations

NSCLC: non-small cell lung cancer
LUAD: lung adenocarcinoma
LUSC: lung squamous cell carcinoma
SIRT3: sirtuin 3
OA: oroxylin A
OA-OEt: oroxylin A phosphate diethyl ester
OA-OH: oroxylin A phosphate derivative
ROS: reactive oxygen species
mtROS: mitochondrial reactive oxygen species
mPTP: mitochondrial permeability transition pore
TEM: transmission electron microscopy
OCR: oxygen consumption rate
ECAR: extracellular acidification rate
IHC: immunohistochemistry
SDS-PAGE: sodium dodecyl sulfate-polyacrylamide gel electrophoresis
PVDF: polyvinylidene fluoride
PI: propidium iodide
DAPI: 4',6-diamidino-2-phenylindole
TUNEL: terminal deoxynucleotidyl transferase dUTP nick end labeling
qPCR: quantitative polymerase chain reaction
DRP1: dynamin-related protein 1
FIS1: fission 1
OPA1: optic atrophy 1
MFN2: mitofusin 2
SOD2: superoxide dismutase 2
NAD+: nicotinamide adenine dinucleotide
