# Supplementary material for: SIRT3 activation by oroxylin A phosphate diethyl ester triggers mitochondrial dysfunction and apoptosis in non-small cell lung cancer
Source: Int J Biol Sci. 2026 May 11;22(10):5283–300. doi: 10.7150/ijbs.133993 (PMC13215253; doi:10.7150/ijbs.133993)
Supplement: Supplementary file 1 — Supplementary figures and tables. [file ijbsv22p5283s1.pdf]

Supplementary Figures

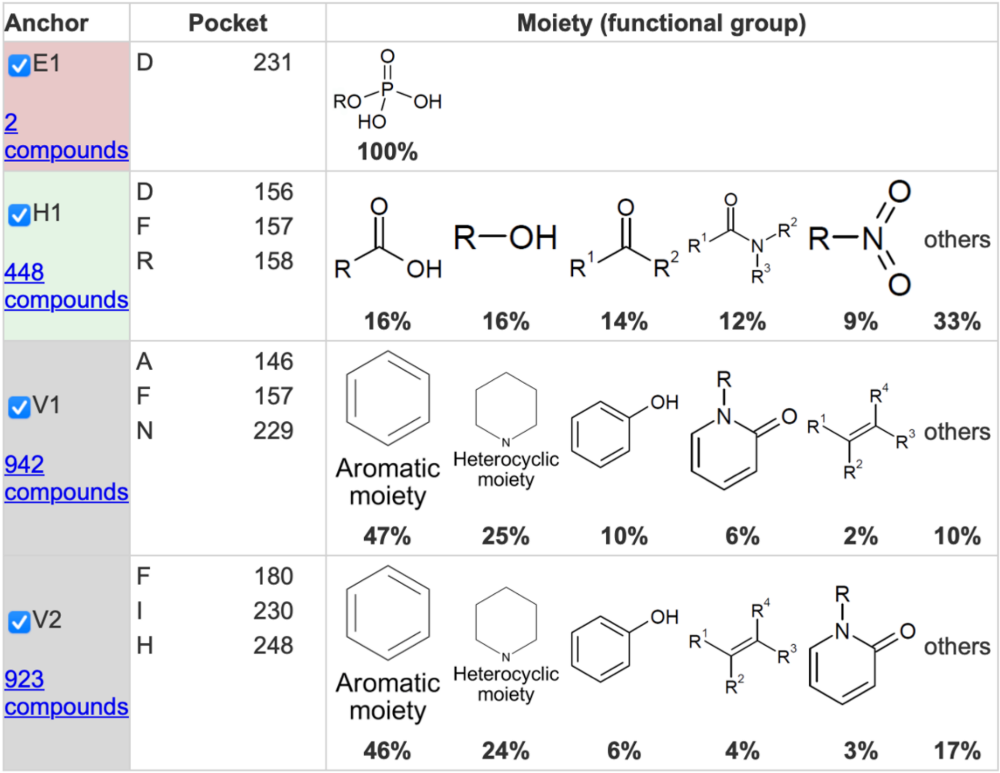

Figure S1. Analysis of the binding pocket by the SiMMap server. The “Anchor” column shows E stands for electrostatic, H for hydrogen bonding, and V for van der Waals forces. The number of compounds indicates how many of the top 1,000 ranked compounds match this anchor. The “Pocket” column shows the important residues contributing to the binding potencies for each anchor. The “Moiety (functional group)” column implies the functional group distribution in each anchor.

A

Hydrophobic Interactions

Index	Residue	AA	Distance	Ligand Atom	Protein Atom
1	157A	PHE	3.28	2504	268
2	157A	PHE	3.43	2515	265
3	199A	LEU	2.85	2495	619
4	230A	ILE	3.77	2504	884
5	248A	HIS	3.37	2510	1012
6	294A	PHE	3.99	2512	1364

Hydrogen Bonds

Index	Residue	AA	Distance H-A	Distance D-A	Donor Angle	Protein donor?	Side chain	Donor Atom	Acceptor Atom
1	156A	ASP	3.38	3.75	105.28	×	✓	2519 [O3]	257 [O.co2]
2	157A	PHE	2.12	3.08	164.53	✓	×	258 [Nam]	2519 [O3]
3	230A	ILE	2.08	2.84	132.31	✓	×	879 [Nam]	2499 [O3]
4	231A	ASP	2.31	2.87	115.13	✓	×	887 [Nam]	2499 [O3]

π-Stacking

Index	Residue	AA	Distance	Angle	Offset	Stacking Type	Ligand Atoms
1	248A	HIS	3.65	17.82	0.29	P	2508, 2509, 2510, 2511, 2512, 2513

B

Hydrophobic Interactions

Index	Residue	AA	Distance	Ligand Atom	Protein Atom
1	154A	ILE	3.72	2495	241
2	230A	ILE	3.67	2501	884
3	294A	PHE	3.76	2512	1364

Hydrogen Bonds

Index	Residue	AA	Distance H-A	Distance D-A	Donor Angle	Protein donor?	Side chain	Donor Atom	Acceptor Atom
1	157A	PHE	2.07	3.01	156.86	✓	×	258 [Nam]	2508 [O2]
2	230A	ILE	2.41	3.12	127.79	✓	×	879 [Nam]	2503 [O2]
3	231A	ASP	2.36	3.05	126.32	✓	×	887 [Nam]	2503 [O2]

Water Bridges

Index	Residue	AA	Dist. A-W	Dist. D-W	Donor Angle	Water Angle	Protein donor?	Donor Atom	Acceptor Atom	Water Atom
1	154A	ILE	2.70	2.64	155.88	81.58	×	2515 [O3]	238 [O2]	2219
2	155A	PRO	3.90	2.64	155.88	136.81	×	2515 [O3]	246 [O2]	2219

π-Stacking

Index	Residue	AA	Distance	Angle	Offset	Stacking Type	Ligand Atoms
1	248A	HIS	5.07	26.90	1.40	P	2509, 2510, 2511, 2512, 2513, 2514

Figure S2. Analysis of interactions between SIRT3 and OA-OEt (A) and OA (B).

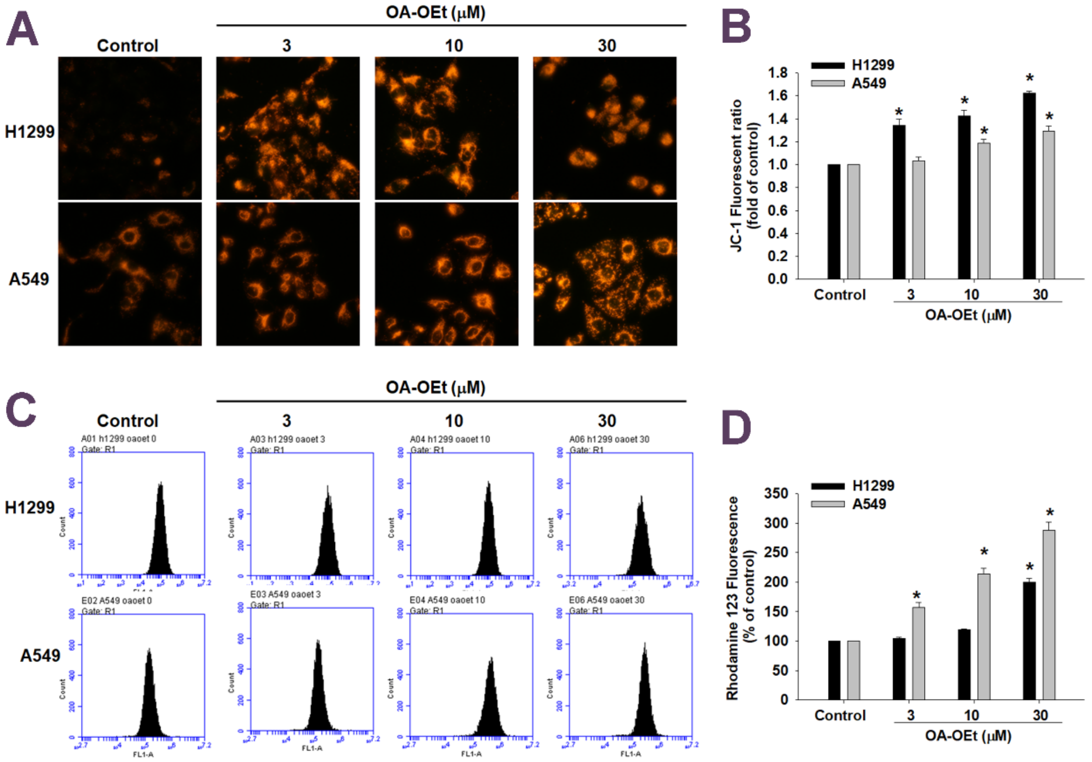

Figure S3. OA-OEt transiently increases mitochondrial membrane potential in H1299 and A549 cells. H1299 and A549 cells were treated with OA-OEt (0, 3, 10, or 30 μM) for 8 h. (A) Representative fluorescence images of JC-1 staining showing increased red fluorescence in OA-OEt-treated cells. (B) Quantification of the JC-1 red/green fluorescence ratio by flow cytometry in H1299 and A549 cells. (C) Representative flow cytometric histograms of Rhodamine 123 fluorescence after OA-OEt treatment. (D) Quantification of Rhodamine 123 fluorescence intensity expressed as percentage of the control. Data are presented as the mean ± SD from four independent experiments (n = 4). **p* < 0.05 compared with the control group.

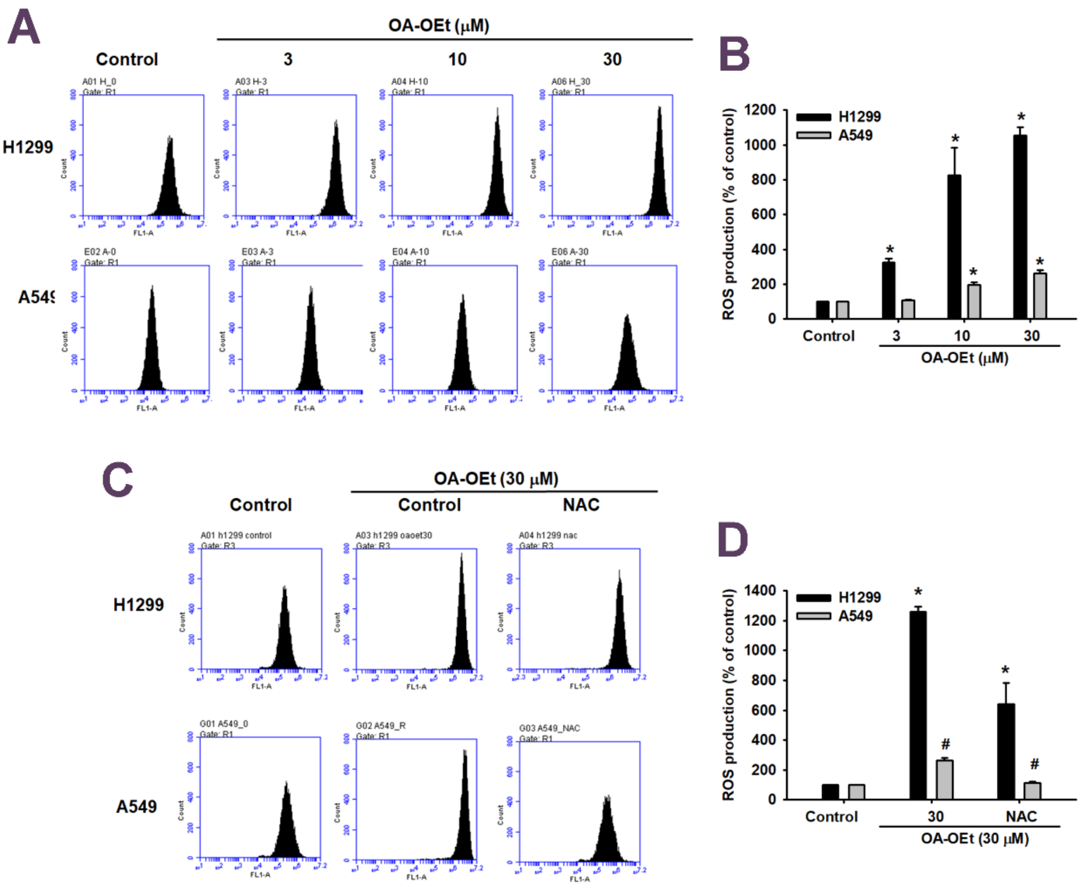

Figure S4. OA-OEt induces intracellular ROS accumulation in NSCLC cells. H1299 and A549 cells were treated with OA-OEt (0, 3, 10, or 30 μM) for 1 h, followed by staining with H2DCFDA (1 μM) and flow cytometric analysis of DCF fluorescence. (A) Representative flow cytometric histograms of H2DCFDA fluorescence in H1299 and A549 cells after OA-OEt treatment. (B) Quantification of intracellular ROS levels expressed as percentage of the control in H1299 and A549 cells. (C) Representative histograms of H2DCFDA fluorescence in cells treated with OA-OEt (30 μM, 1 h) with or without NAC pretreatment. (D) Quantification of ROS levels in the presence or absence of NAC. Data are presented as the mean ± SD from four independent experiments (n = 4). *$p < 0.05$ versus control; # $p < 0.05$ versus OA-OEt (30 μM) alone.
